# The management of stress urinary incontinence: A case report

**DOI:** 10.4102/sajp.v71i1.229

**Published:** 2015-05-29

**Authors:** Preshani Reddy

**Affiliations:** 1 Department of Physiotherapy, University of KwaZulu-Natal, Westville Campus, South Africa

## Abstract

**Introduction:**

Conservative management is the first option for patients with stress urinary incontinence (SUI). However, successful management of women diagnosed with SUI is dependent on a proper assessment and a tailored treatment plan. This case report aims to show the effectiveness of physiotherapy management in a 42-year-old patient diagnosed with SUI.

**Patient presentation:**

The patient’s main complaints were involuntary loss of urine on coughing, sneezing and lifting of heavy objects, which started following the birth of her third child.

**Management and outcome:**

The patient was taught the ‘Knack’ manoeuvre and provided with a tailored pelvic floor exercise programme. Improvement was noted at the third visit and the patient no longer had involuntary episodes.

**Conclusion:**

This case report shows the successful outcome of conservative management in a patient with stress urinary incontinence.

## Introduction

Stress urinary incontinence (SUI) is the most common subset of urinary incontinence affecting women (Elmissiry, Mahdy & Ghoniem 2011). Globally, literature estimates 200 million women live with incontinence (Norton & Brubaker 2006). However, the prevalence may be higher as many females with SUI do not seek help for fear of embarrassment (Luber 2004). Stress urinary incontinence as defined by Haylen *et al.* (2010:5) is a ‘complaint of involuntary loss of urine on effort or physical exertion (e.g. sporting activities), or on sneezing or coughing’. Physiologically it occurs as a result of a sudden increase in intra-abdominal force resulting in the bladder pressure exceeding the urethral pressure (Norton & Brubaker 2006).

Factors that predispose women to SUI, such as ageing, smoking and obesity, have been highlighted in literature (Luber 2004). In terms of pregnancy and childbirth there has been conflicting evidence and the influence of these factors is not well understood (Luber 2004). However, a study by Lukacz *et al.* (2006) revealed that women who delivered vaginally had a higher risk for pelvic floor disorders, which is independent of parity. Furthermore, women who sustain anatomical or neuromuscular changes following childbirth may remain asymptomatic as a result of compensatory mechanisms (Bump & Norton 1998) and only display signs of SUI later on because of muscle loss as a result of ageing or injury (Norton & Brubaker 2006). The management of SUI does vary but conservative management should initially be considered (National Institute for Health Clinical Excellence [NICE] 2013). There is presently limited literature nationally on the effectiveness of physiotherapy management in women with SUI. This case report will therefore aid in contributing to the evidence required in this under-researched area. The present case report describes a 42-year-old woman with possible SUI who was referred by a gynaecologist.

## Ethical approval

Written patient consent was obtained prior to publication of this case history.

## Patient presentation

### Subjective assessment

Mrs X reported that her problem started 5 months earlier, following the birth of her third child. She stated that she wet herself if she sneezed or coughed and had reduced her fluid intake in an effort to prevent this from occurring. She also urinated more frequently to ensure her bladder is empty. When her symptoms persisted, she made an appointment with her gynaecologist, who referred her to physiotherapy for management. Further questioning of her bladder symptoms revealed that she had started wearing a panty-liner. She stated that if she has to cough she ‘wees’ a few drops (but only if she coughs hard) and every time she sneezes she ‘wees’ a few drops, which is worse if her bladder is full. She had also noticed a few leakage episodes when she lifted her 2-year-old daughter (10 kg). She stated that she wet herself about twice a day and changed her panty-liner three times daily.

The patient had no relevant bowel problems and was menstruating normally. With regard to her obstetric and gynaecological history, all her deliveries were vaginal with no perineal injury and no urinary incontinence (UI) after the birth of her previous two children. The patient did not have any positive red flags as defined by the NICE guidelines (NICE 2005, 2011, 2013). Her last pap smear prior to her pregnancy was normal. There were no relevant previous surgeries noted. The patient was not taking any medication at the time and had no chronic lifestyle diseases. She did not smoke and consumed alcohol occasionally, but not in the 18 months prior to her assessment. The visual analogue scale was used to determine the effect of her UI on her quality of life. With 0 being not affected and 10 being highly affected, she scored herself a 6.

The patient was working as a secretary in a legal firm at the time. She did not exercise because of time constraints.

Following completion of the subjective assessment, a preliminary hypothesis of SUI was made. The patient’s history was in keeping with SUI symptoms, as outlined by the Haylen *et al.* (2010). A basic explanation of the pelvic anatomy and physiology aided by diagrams, which is an aspect of behavioural therapy (Rovner & Wein 2004), was given to the patient. Following the explanation, a three-day bladder diary was given to the patient to complete in order to establish her voiding habits and caffeine intake (NICE 2013). Although her symptoms are strongly associated with SUI, Fink *et al.* (1999) stated that nocturnal micturition is a primary parameter in a bladder diary for differentiating between urge and stress urinary incontinence. Additionally, using a combination of subjective and objective self-resignation may improve diagnostic ability (Fink *et al.* 1999). The importance of accurate recording was explained to the patient, who was subsequently requested to repeat the instructions to ensure correct understanding. The patient was then made aware that the follow-up appointment would include an internal vaginal examination; the reasons were clearly explained to the patient (Chartered Society of Physiotherapy [CSP] 2002). The patient was mentally competent and provided verbal consent for the examination, which was documented in the patient’s file (CSP 2005). The patient was allowed to bring a chaperone (which she declined) and was given the option to tell the therapist to stop at any point during the examination, should she feel uncomfortable. The patient’s next appointment was scheduled for 1 week later.

### Objective assessment

At session two, the bladder diary of the patient was analysed and the following was found:

- Average number of voids: 3
- Average number of accidental episodes: 2
- She changed her panty-liner 3 times per day
- Maximum volume voided: 300 mL
- Minimum volume voided: < 50 mL
- No night-time micturition.

The patient’s Body Mass Index was within normal range (23). She was made comfortable prior to the examination and the procedure was briefly explained again. The therapist followed the infection control procedure as per protocol.

#### Findings on examination

On initial examination the following was noted:

- no abdominal tenderness or bloating was noted
- no perineal abnormalities were present, that is, irritation or redness in the area
- a mediolateral episiotomy was seen; the scar was small, healed and mobile
- she had no dermatomal abnormalities at S2–4
- she had perineal descent
- cough: leakage noted.

The PERFECT scheme was used to determine the muscle function of the patient. The tool is a reliable and valid method of assessing pelvic floor function (Laycock, Whelan & Dumoulin 2008). The tool was also selected as it provides a baseline measure for the patient and assists when planning a patient-specific exercise (Laycock & Jerwood 2001). The patient’s PERFECT score was determined on the 7th contraction (Table 1).

**TABLE 1:** Participant’s PERFECT score on assessment.

Variable	Initial assessment	3-week follow-up	1-month follow-up
Strength of contraction (P)	3	3	4
Length of hold (seconds) (E)	5	7	9
Repetitions (number) (R)	7	8	8
Fast contractions (number) (F)	4	6	9
Elevation during contraction (E)	Yes	Yes	Yes
Co-contraction of transverse abdominis (C)	Yes	Yes	Yes
Coordination of contraction prior to cough (T)	No	Yes	Yes

A final diagnosis of SUI was established based on the objective examination findings. The bladder diary revealed that the patient had a high micturition and low volume rate. This was coupled with leakage episodes that were brought upon in instances of physical exertion (lifting her child) and sudden increases in intra-abdominal pressure (coughing or sneezing). She also reported only two episodes of ‘urge’ UI. These symptoms are in keeping with SUI, as outlined by Haylen *et al.* (2010) and Parsons *et al.* (2007).

Based on the vaginal examination, her muscle strength was a Grade 3. She was unable to time her muscle contraction and her cough that resulted in her leakage. A brief explanation of the above was given to the patient and the patient was also instructed that she needed to work on her endurance and the timing of her fast contractions. It was explained to the patient that compliance with the pelvic floor muscle exercises would aid in preventing accidental leakages.

## Management and outcome

The following tailored treatment plan was discussed with the patient:

- the patient was taught to perform the ‘Knack’ manoeuvre prior to strenuous activities such as lifting, coughing or sneezing
- 4 × fast 1-second contractions; 3 × daily
- 7 × 5-second-hold contractions; 3 × daily.

### Exercise progression

The progression from the exercise follows the principles set out by Laycock and Jerwood (2001), namely:

- gradually increase the number of fast contractions
- increase the number of repetitions of long maximum voluntary contractions until 10 is reached
- gradually increase the hold of the contractions until 10 seconds is reached (therefore reaching the ideal 10 contractions x 10-second hold).

This was accomplished gradually over 3 months (NICE 2013). The exercises were written down for the patient.

Lifestyle advice was given to correct voiding habits as she was voiding ‘just in case’ to prevent accidents. The patient was asked to slowly increase the time between voiding (15–30 minutes) until she reached an acceptable voiding interval of 2–4 hours (Rovner & Wein 2004). A three-week appointment followed by monthly follow-up appointments were made to ensure appropriate progression of the exercises and that she was contracting her pelvic floor muscles correctly. The patient showed improvement at the three-week and one-month follow-up sessions (Table 1) and her exercises were adjusted accordingly. At the second visit, the patient rated the impact of SUI on her quality of life as 4. She successfully increased time between voiding to 75 minutes and had three separate involuntary episodes, which she expressed as ‘a few drops’. At the third visit, the patient rated the effect of her UI on her quality of life as 1; she had no episodes and had reached a voiding interval of 2 hours. Mrs X did not return for a follow-up visit but stated that she no longer had any complaints.

## Discussion

Conservative management is the first option for patients with SUI (NICE 2013). Behavioural therapy in the form lifestyle advice is a necessary aspect of treatment (Rovner & Wein 2004; NICE 2013). Whilst there is no evidence to show an association between drinking tea and SUI (Jura *et al.* 2011), the patient voided after every cup of tea. The therapist therefore suggested that she change to decaffeinated tea as caffeine does have a diuretic effect (Jura *et al.* 2011) and increase her voiding intervals, which was successfully accomplished. A vaginal examination was conducted, as recommended by NICE guidelines (2013), prior to supervised pelvic floor muscle training. The use of biofeedback and electrical stimulation for treatment is not routine (NICE 2013) but would have been considered to facilitate pelvic floor contractions should she have failed or struggled to isolate the muscles independently. The ‘Knack’ manoeuvre (Miller, Ashton-Miller & DeLancey 1998) is performed by consciously contracting the pelvic floor muscles prior to physical exertion which results in an increase in intra-abdominal pressure (i.e. coughing or sneezing) and maintaining the contraction for the duration of the stress. This manoeuvre is thought to prevent the urethra and bladder base descending (Price, Dawood & Jackson 2010) and improve urethral closing pressure, thus increasing continence (Miller *et al.* 2008). A combination of the ‘Knack’ manoeuvre and a strength training exercise, which was found to be successful in treating this patient, is effective in pelvic floor muscle training (Bø 2004). Literature in this area has also found that these ‘two systems have a common base in the pelvic floor acting as a structural base, stabilizing the bladder and urethra during increases in abdominal pressure’ (Bø 2004:82). The pelvic floor muscles are composed of striated muscle; therefore designing the treatment programme for pelvic floor muscles follows the same principles as for striated muscle (Laycock & Jerwood 2001; Price *et al.* 2010). NICE guidelines (2013) suggest an exercise programme consisting of at least eight contractions three times a day for a minimum of 3 months, as a first-line treatment for urinary incontinence. However, Laycock and Jerwood (2001) suggest translating the findings from the PERFECT tool into a patient-specific exercise regimen, as was done in this case report. In addition, Laycock and Jerwood (2001) state that this would satisfy the muscle training principles of overload and specificity and possibly improve patient compliance. The use of pharmacological drugs such as duloxetine was not considered, as it is not a first-line treatment method (NICE 2013). However, it would have been recommended if the patient showed no signs of improvement with conservative therapy and did not want surgery (NICE 2013).

## Conclusion

Conservative management is the first option for patients with SUI. This case report shows the successful management of a patient diagnosed with SUI following a patient-specific physiotherapy treatment plan. Although the results cannot be generalised, this case report highlights the importance of assessment and the effectiveness of physiotherapy in managing a patient with SUI.
